# Supplementary material for: Empirical methods for controlling false positives and estimating confidence in ChIP-Seq peaks
Source: BMC Bioinformatics. 2008 Dec 5;9:523. doi: 10.1186/1471-2105-9-523 (PMC2628906; doi:10.1186/1471-2105-9-523)
Supplement: Additional File 1 — A variety of html documents from the USeq web site detailing the available applications, their best usage, output file type descriptions, command line menus, etc. [file 1471-2105-9-523-S1.zip › USeqUserGuides/JavaDoc.zip!/JavaDoc/util/bio/digest/package-use.html]

Uses of Package util.bio.digest


|  |  |  |
| --- | --- | --- |
| **Overview** \| **Package** \| Class \| **Use** \| **Tree** \| **Deprecated** \| **Index** \| **Help** | |  |
| PREV   NEXT | **FRAMES**    **NO FRAMES**     **All Classes** |  |


---


## **Uses of Package util.bio.digest**

| Packages that use util.bio.digest | |
| --- | --- |
| **bioroot.oligo** |  |
| **bioroot.plasmid** |  |
| **util.bio.digest** | Restriction enzyme digestion of DNA sequences. |

Classes in util.bio.digest used by bioroot.oligo

****Enzyme****
  
          Restriction enzyme information.

Classes in util.bio.digest used by bioroot.plasmid

****Enzyme****
  
          Restriction enzyme information.

Classes in util.bio.digest used by util.bio.digest

****Enzyme****
  
          Restriction enzyme information.

---


|  |  |  |
| --- | --- | --- |
| **Overview** \| **Package** \| Class \| **Use** \| **Tree** \| **Deprecated** \| **Index** \| **Help** | |  |
| PREV   NEXT | **FRAMES**    **NO FRAMES**     **All Classes** |  |


---
